# The regulation of working memory by rTMS target on cerebellum with different frequencies in healthy adults

**DOI:** 10.1002/alz.088237

**Published:** 2025-01-03

**Authors:** Bo Song, Jingping Shi, Minjie Tian

**Affiliations:** ^1^ The Affiliated Brain Hospital of Nanjing Medical University, Nanjing, Jiangsu China

## Abstract

**Background:**

The study of the involvement of the cerebellum in learning and memory has become one of the recent hot topics in the field of cognitive neuroscience. Transcranial magnetic stimulation (TMS) of the cerebellum has gained increasing interest in the treatment of cognition‐related disorders, making it necessary to determine the optimal parameters for cerebellar TMS. In this study, we aim to explore the effects of different frequencies of cerebellar repetitive TMS (rTMS) on working memory regulation and the associated electrophysiological changes. Through this research, we hope to provide objective bioinformatic guiding evidence for parameter optimization of cerebellar TMS.

**Method:**

We recruited 75 healthy university students or graduate students as participants and divided them into five groups: 1Hz rTMS group, 5Hz rTMS group, 10Hz rTMS group, 20Hz rTMS group, and sham stimulation group. Each group received rTMS at different frequencies (1Hz, 5Hz, 10Hz, and 20Hz) over the cerebellar Crus II region, while a control group received sham stimulation. We assessed working memory performance using a 2‐back task and recorded electroencephalographic (EEG) signals during the 2‐back task and resting state before and after the stimulation. Cluster‐based permutation statistics and One‐way analysis of variance were used for the analysis of both neurophysiological and behavioral data.

**Result:**

The 5Hz rTMS significantly increased the induced ERP activity of N100, P150, and N200, showed greater theta and alpha oscillatory activity, and significantly improved working memory performance. Furthermore, using phase locking value to construct brain networks and conduct analysis, we found that 5Hz rTMS significantly enhanced the whole‐brain local and global efficiency of brain networks in the theta frequency band, while 20Hz rTMS improved the local efficiency of the occipital and parietal lobes in the beta frequency band, with no significant differences observed in other frequency bands.

**Conclusion:**

Our results demonstrate that 5Hz rTMS stimulation can significantly improve subjects' neurophysiological changes and working memory performance, showing a certain frequency‐dependent effect. These findings contribute to providing scientific evidence for the application of cerebellar TMS in memory improvement and offer valuable references for future related research.

